# Patient preferences for adherence to treatment for osteoarthritis: the MEdication Decisions in Osteoarthritis Study (MEDOS)

**DOI:** 10.1186/1471-2474-14-160

**Published:** 2013-05-06

**Authors:** Tracey-Lea Laba, Jo-anne Brien, Marlene Fransen, Stephen Jan

**Affiliations:** 1 Faculty of Pharmacy, The University of Sydney, Camperdown, Sydney, Australia; 2 The George Institute for Global Health, Camperdown, Sydney, Australia; 3 St Vincent’s Hospital, Darlinghurst, Sydney, Australia; 4 Faculty of Medicine, The University of New South Wales, Kensington, Sydney, Australia; 5 Faculty of Health Sciences, The University of Sydney, Lidcombe, Sydney, Australia; 6 School of Medicine, The University of Sydney, Camperdown, Sydney, Australia

**Keywords:** Osteoarthritis, Discrete choice experiment, Intentional medication adherence

## Abstract

**Background:**

Often affecting knee joints, osteoarthritis (OA) is the most common type of arthritis and by 2020 is predicted to become the fourth leading cause of disability globally. Without cure, medication management is symptomatic, mostly with simple analgesics such as acetaminophen and non-steroidal anti-inflammatory drugs (NSAIDs), and glucosamine sulfate. Adherence to arthritis medications is generally low. Intentional non-adherence, that is deliberate decision-making about the use of analgesics, occurs in OA patients. To date, a limited number of studies have explored medication-taking decisions in people with OA nor the extent to which individuals’ trade off one treatment factor for another in their decision-making using quantitative techniques. This study aimed to estimate the relative influence of medication-related factors and respondent characteristics on decisions to continue medications among people with symptomatic OA.

**Methods:**

A discrete choice experiment (DCE) was conducted among participants attending end-of-study visits in the **L**ong-term **E**valuation of **G**lucosamine **S**ulfate (LEGS) study (*ClinicalTrials.gov* ID: NCT00513422). The paper-based survey was used to estimate the relative importance of seven medication specific factors (pain efficacy, mode of action, dose frequency, treatment schedule, side effects, prescription, and out-of-pocket costs) and respondent characteristics on decisions to continue medications.

**Results:**

188 (response rate 37%) completed surveys were returned. Four of the seven medication factors (side effects, out-of-pocket costs, mode of action, treatment schedule) had a significant effect on the choice to continue medication; patient characteristics did not. Assuming equivalent pain efficacy and disease-modifying properties for glucosamine, the positive relative likelihood of continuing with sustained-release acetaminophen was equivalent to glucosamine. By contrast, the negative relative likelihood of NSAID continuation was mostly driven by the side effect profile. The predicted probability of continuing with glucosamine decreased with increasing out-of-pocket costs.

**Conclusions:**

This study has characterised the complexity of medication-taking decisions that potentially underpin intentional non-adherent behaviour for people with symptomatic OA. In particular, medication risks and cost were important and ought to be borne into considerations in interpreting clinical trial evidence for practice. Ultimately addressing these factors may be the way forward to realising the full potential of health and economic benefits from the efficacious and safe use of OA medications.

## Background

Osteoarthritis (OA) is a musculoskeletal disease that causes chronic joint pain and reduced physical functioning. Often affecting knee joints, OA is the most common type of arthritis. By 2020, OA is predicted to become the fourth leading cause of disability globally [1].

Currently there is no known cure for OA, nor are there effective interventions to slow disease progression [2-4]. Medication management is symptomatic, mostly with simple analgesics such as acetaminophen and non-steroidal anti-inflammatory drugs (NSAIDs) [5,6]. Increasingly, glucosamine sulfate (GS) [7], is being used as a potential analgesic and disease-modifying agent [3,8-11]. In Australia, GS is considered a dietary supplement and is purchased without prescription. Unlike other OA medications, the cost of GS is not subsidised by the Australian government [12].

As occurs with most chronic conditions, adherence to arthritis medications is low [4,13-16]. Factors implicated in adherence to OA and other anti-rheumatic medications include dosing frequency [16], pain and self-efficacy levels [13], and physician trust [4,17,18]. Intentional non-adherence [19], that is deliberate decision-making about the use of OA medications, is reported in the literature. In particular, intentional under-dosing and rationing of analgesics occurs [20-22]. Such decisions appear to be driven by factors including the fear of addiction [20], previous medication effectiveness, and the burden and illness stigma represented by increased pill loads [21]. For NSAIDs specifically, a high level of trust in the prescribing physician influences decisions [22].

Primarily, qualitative methods have been used to investigate medication decisions in OA. Although a limited number of studies have used quantitative techniques, the extent to which individuals’ trade off one treatment factor for another in decision-making about medication adherence has not been extensively studied [23-28]. Physicians and policy makers could use such information to tailor adherence support to match the preferences of OA patients.

Discrete choice experiment (DCE) is a survey methodology that can be used to elicit preferences to quantitatively determine the relative influence of factors on decision-making with regard to medication adherence [29,30]. Developed initially in marketing research, DCEs are used increasingly in health economics and are considered state-of-the-art in this field to elicit preferences for health services [31-35]. To date, three DCE studies have explored patient preferences for treatment factors associated with knee and/or hip osteoarthritis [23,24,28]. In these studies, both efficacy and the gastrointestinal side effects of treatment significantly impacted patient choice. However, other factors potentially relevant to OA medication adherence were not consistently included. In particular, neither the cardiovascular, hepatic or renal side effects nor the chronic or intermittent treatment scheduling of OA therapy were incorporated. Additionally, preferences about acetaminophen were omitted from one study [24].

In Australia, the LEGS (**L**ong-term **E**valuation of **G**lucosamine **S**ulfate) study was a two-year, double-blind, placebo-controlled randomised clinical trial aiming to evaluate whether the dietary supplements, GS and/or chondroitin can limit or reduce structural disease progression (cartilage loss), whilst providing pain relief, in people with osteoarthritis of the knee (ClinicalTrials.gov Identifier NCT00513422). Throughout the LEGS study, as is typically the case in clinical trials, a number of trial-related factors could potentially affect treatment decisions and adherence outside of the trial setting. Firstly, as a part of the study protocol, participants were regularly encouraged to persist with the study treatments, even in the absence of knee pain. Furthermore, study treatments were mailed to participants and provided free of charge. The use of DCEs potentially helps understand the effects of such factors, including out-of-pocket costs, beyond the clinical trial setting.

The **Me**dication **D**ecisions in **O**steoarthritis **S**tudy (MEDOS) aimed to estimate the relative influence of different medication-related factors and respondent characteristics on decisions to continue medications among people with symptomatic OA.

## Methods

A paper-based survey was given to all LEGS participants attending their end-of-study visit by a member of the LEGS research team; surveys were mailed to participants who had already completed end-of-study visits at MEDOS commencement. The survey was self-administered and completed either during the end-of-study visit or at a later date and returned via mail.

### Survey instrument

The survey used a DCE approach and comprised 16 hypothetical choice tasks Additional questions about self-reported adherence to study treatment and other prescribed medications during the LEGS study [36], and an eight-item scale from the primary care assessment survey (PCAS) [37] were also included. The PCAS has been used in other adherence-related research [36], and assesses the level of trust held by a patient for his/her provider.

### Instrument development

In a DCE, respondents are offered a series of hypothetical pairwise alternatives (choice set), and asked to nominate the preferred alternative. Each alternative is described by a set of factors with pre-specified levels. The levels assigned to each alternative are varied successively across each choice set [32,38,39]. For this study, factors were identified through literature review and with respect to the currently available OA treatments in Australia [3,12]. Further factor refinement occurred through survey piloting among healthcare professionals and a general population experienced with analgesic use. Seven factors considered most important through survey pre-testing and used in the final survey are summarised in Table 1, which also includes description of the levels of each factor. Extensive descriptions of the factors were included in the survey introduction. Participants were advised to contact the research team should assistance be required.

**Table 1:** Description of factors and levels used in the discrete choice experiment

**Factor**	**Description**	**Levels**
**Pain Efficacy**	What the pain can be reduced to (from 9/10)	1, 3, 4, 7
**Mode of action**	How the medication works	Quick pain relief (Base)
		Slow Osteoarthritis
**Dose frequency**	How often taken per day	1, 3 (Base)
**Treatment Schedule**	How regularly taken	When needed (Base)
		Daily
**Cost**	Cost to YOU every month	$AUS 5, 20, 35 50
**Prescription**	Prescription/purchase restrictions	Yes: (pharmacy **with** prescription)
		No: (pharmacy, health food store or supermarket **without** prescription) (Base)
**Side effects**	Possible side effects of the medication	No side effects (Base)
		Drowsy/constipated
		Heartburn/reflux, stomach ulcers
		High blood pressure, heart/kidney/liver problems

The final survey included 16 choice sets. In each, respondents were presented with hypothetical medication alternatives, ‘Medication A’ and ‘Medication B’. To eliminate product recognition bias, brand names were not used. Respondents were asked to imagine their current pain score (as measured throughout the LEGS study) was 9 out of 10 and they were currently taking both tablets, of which their doctor was aware. Given a choice between the two medication options, respondents were asked to indicate which medication they would prefer to continue taking.

On the basis of the factors and levels listed in Table 1, an orthogonal design was generated using the choice experiment design software Ngene Version 1.0 [40]. Two survey versions were created by randomly ordering the choice sets using a random number generator [41]. The survey was pilot tested (n = 5) to check for any problems with interpretation and face validity; only minor changes to the layout were made.

### Participants

All LEGS participants completing their end-of-study visit were eligible to participate in MEDOS. The eligibility criteria for participation in the LEGS study can be found in Additional file 1[42].

The University of Sydney Human Research Ethics Committee (HREC 8821, amendment 4th May 2010) and the Royal Australian College of General Practitioners (NREEC 06/006, amendment 18th June 2010) approved this study.

### Analysis

The background characteristics of MEDOS participants were summarised and compared with all LEGS participants attending their final follow-up visit. Additionally, self-reported adherence to study treatment, cost-related non-adherence in the past 12 months, and the transformed PCAS Physician Trust domain (maximum score 100) were summarised for MEDOS participants.

For the choice data, a panel mixed multinomial (random parameters) logit (MMNL) model was used [32,38] to investigate changes in utility (U) (i.e. preference to continue taking a medication) when the level of a factor was changed using NLOGIT Version 4.0. A higher or more positive utility indicates increased preference to continue a medication. Additional file 2 details the model form and analysis plan.

The effect on the final model of respondent characteristics was investigated by forward stepwise addition followed by backwards elimination of significant covariates. A differential out-of-pocket cost factor was investigated based on work status (employed, unemployed, retired/semi-retired), as well as healthcare concession card or private health insurance through the incorporation of cost-factor interaction terms.

From the final model, the odds ratio (OR) of each factor was calculated (i.e. OR = exp(*β*)), representing the influence of the factor on the choice to continue a medication. Odds ratios were also calculated for the medication profiles for GS, sustained-release acetaminophen, and selective and non-selective NSAIDs by inputting the factor levels outlined in Table 2. This represents the relative likelihood of continuing each medication profile: an OR greater than 1 represents an increased preference to continue taking medication.

**Table 2:** Factor Levels used in calculating odds ratios and predicted probabilities

**Factor**	**Glucosamine**	**Acetaminophen Sustained release**	**NSAID (selective, e.g. celecoxib)**	**NSAID (non-selective, e.g. ibuprofen)**
Pain Efficacy	1	1	1	1
Mode of Action	Slow OA	Quick	Quick	Quick
Dose Frequency	Three	Three	Once	Three
Treatment Schedule	Daily	Daily	Daily	Daily
Cost ($AU)^a^	$20	$12.00	$34.20	$20
Prescription	No	No	Yes	No
Side effects	Nil	Nil	High blood pressure, heart/kidney/liver problems	Heartburn/reflux, stomach ulcers

^a^ The out-of-pocket costs represent those that would have been incurred at the time of the survey for non-healthcare concession cardholders.

The willingness to accept (WTA) for each factor was estimated by taking the marginal rate of substitution between the factor and cost (*β*_*factor*_/*β*_*cost*_) [43]. This describes the amount of money respondents believe compensates for a given change in the factor.

The relative importance of factors and their levels was also investigated [34]. This reflects the extent to which the difference between the best and worst levels of each characteristic drives the decision to continue taking a medication.

Finally, the predicted probability of continuing GS was calculated using the factor levels outlined in Table 2[44]. As the cost of GS is variable within Australia, a cost sensitivity analysis was conducted for the predicted probability.

## Results

503 LEGS participants attended the end-of-study visits; 59 participants had already attended the end-of-study visit at MEDOS commencement and were mailed a survey. The remaining participants were given a survey at the end-of-study visit.

188 (response rate 37%) completed surveys were returned. Table 3 displays the background characteristics for all LEGS participants attending their end-of-study visit, and the subset of these participants completing the MEDOS study. With the exception of a lower proportion of people taking “when required” medications in the MEDOS group, there does not appear to be evidence for difference.

**Table 3:** Background characteristics

	**Responders (n = 188)**	**Missing (n)**	**All (n = 503)**	**Missing (n)**
Age (mean, SD), years	62, 8.5	15	62, 8	1
Gender (n, %), male	84 (48%)	15	230 (46%)	1
Private Health Insurance (n, %)	108 (62%)	13	308 (61%)	0
Health Care Concession (n, %)	18 (10%)	13	56 (11%)	0
Co-morbidity with treatment (n,%)				
Hypertension or Heart Disease	90 (52%)	15	213 (45%)	1
Ulcer or Stomach Disease	13 (7.5%)	15	46 (9%)	1
Kidney or Liver Disease	0 (0%)	15	4 (1%)	1
Symptom duration (≤5 years) (n, %)	92 (52%)	13	281 (56%)	0
WOMAC Pain (mean, sd)	4.1 (3.6)	0	4.2 (3.5)	0
WOMAC Physical (mean, sd)	15.9 (14.0)	0	17.0 (13.3)	0
Global assessment (mean (SD))	1.6 (1.0)	18	1.8 (1.1)	48
SF12 MCS (mean, SD)	52.7 (10.3)	13	53.5 (9.4)	0
SF12 PCS (mean, SD)	46.3 (9.4)	13	44.4 (9.5)	0
Glucosamine/chondroitin prior (n, %)	61 (35%)	13	154 (31%)	0
Current < daily/when required medication use	5 (3%)	13	38 (8%)*	0
Adherence (study treatment) < 100%^a^	86(53%)	3	N/A^b^	N/A^b^
Cost-related non-adherence^a^	19 (10%)	4	N/A^b^	N/A^b^
Physician Trust^a^ (median, range)	75 (28–100)	0	N/A^b^	N/A^b^

* Sig P < 0.05 ^a^Measured in the MEDOS study only ^b^*N*/*A* not applicable.

For the MEDOS participants, self-reported adherence to the study treatment throughout the LEGS study was generally high, (47% reporting 100% adherence). The lowest adherence rate reported was 75%. For other medications, 24% of participants reported intentionally stopping or altering the dose during the previous 12 months. In general, MEDOS study participants had a high level of trust in their primary care physician (median PCAS score 75 out of 100).

### DCE results

Table 4 shows the results of the DCE. An estimated OR of 0.90 implies that changing the treatment schedule from “when required” to “daily treatment” decreases the likelihood of continuing a medication by 10%, if all other factors are held equal. Likewise, an OR of 0.09 implies that changing from a medication with no side effects to one that may cause high blood pressure, heart, kidney, or liver side effects decreases the likelihood of continuing a medication by 91%.

**Table 4:** Discrete choice experiment results

**Factor**		**OR**^**a **^**(95% CI)**		**RI**^**a**^	**WTA**^**a **^**(95%CI) ($AUD)**	
**Pain Efficacy**^**b **^**(decreasing)**		1.00 (0.97-1.04)		N/A^**a**^	0.08 (0.04-0.11)	
**Mode of action**^**c **^**(Slow OA**^**a**^**)**		1.17 (1.09-1.25)*		5	13.83 (13.73-13.93)	
**Dose frequency**^**b **^**(once/day)**		1.02 (0.96-1.08)			0.58 (0.42-0.75)	
**Treatment Schedule**^**c **^**(daily)**		0.90 (0.89-0.9)*		4	4.11 (3.92-4.29)	
**Cost**^**b **^**(increasing)**		0.97 (0.97-0.98)*		2	N/A^**a**^	
**Prescription**^**b **^**(Yes)**		1.03 (0.97-1.09)		N/A ^**a**^	1.00 (0.83-1.16)	
**Side effects**						
Drowsy/constipated^**c**^		1.55 (1.48-1.62)*		6	18.06 (17.90-18.23)	
Heartburn/reflux, stomach ulcers^**c**^		0.76 (0.75-0.78)*		3	10.67 (10.55-10.78)	
High blood pressure, heart/kidney/liver problems^**c**^		0.09 (0.08-0.11)*		1	90.50 (89.38-91.62)	
**Constant**^**b **^**(α)**		N/A^**a**^		N/A^**a**^		
**Model Fit Statistics**	**Log Likelihood**	−1271	**McFaddens ρ2 adjusted**	0.37	AIC^**a**^	0.869

*P < 0.001.

^a^: *OR* Odds Ratio, *RI* Relative Importance, *WTA* Willingness to Accept, *OA* Osteoarthritis, *N*/*A* not applicable, *AIC* Akaike’s Information Criterion.

^b^: Non-random parameter c: Random parameter.

Four of the seven factors had a significant effect on the choice to continue a medication: out-of-pocket costs, side effects, mode of action, and treatment schedule. Pain efficacy, dose frequency, and whether one’s access to the medication was restricted through prescription and place of purchase did not significantly influence medication choice. The signs of all significant parameters were in the expected direction except for the side effect of drowsiness and constipation, which was positive. A significant constant term (α) indicates that other unmeasured factors considered by respondents, but not included in this experiment, influenced patient decision-making.

Inputting background characteristics into the model did not improve the model fit, nor were the associated β-parameters significant. The relative influence of cost on medication choice was not influenced by health care concession card status, private health insurance status, or work status.

The WTA for each factor is displayed in Table 4. Respondents were willing to accept high blood pressure, heart/kidney/liver problems as a side effect if compensated with up to $92 per month. By contrast, respondents were willing to accept up to $14 per month for a treatment that would only provide pain relief, in comparison to one that would slow OA.

The relative importance of the statistically significant factors is displayed in Table 4. The side effect of high blood pressure, heart/liver/kidney problems was most important, followed by out-of-pocket costs. Respondents were least concerned about the side effect of drowsiness and constipation.

**Figure 1:**
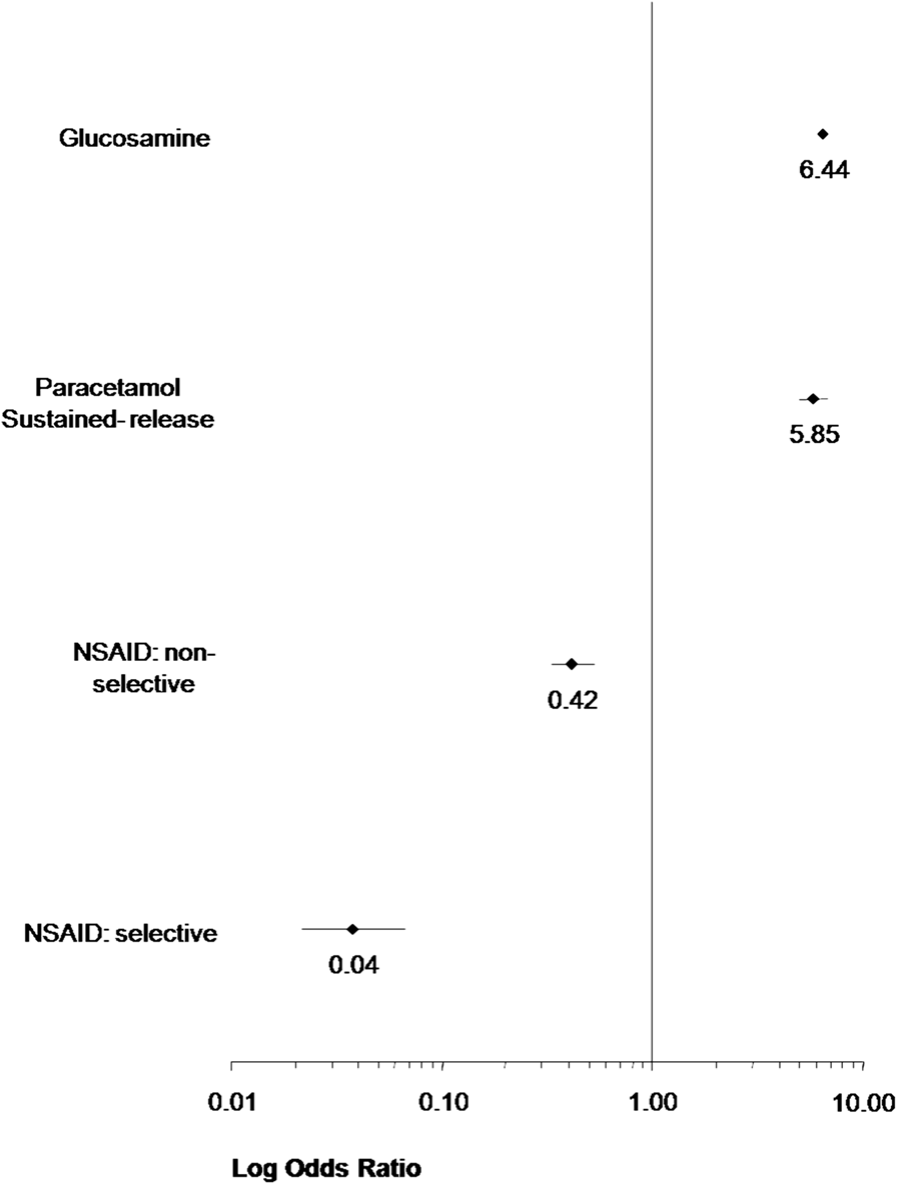
Relative likelihood of continuing a medication.

Figure 1[45] compares the relative likelihood of continuing GS, sustained-release acetaminophen, and selective and non-selective NSAIDs. In this figure, an odds ratio of 1 equates to an average utility (U) of zero, implying that there is no preference (either positive or negative) to continue taking that medication. Assuming equivalent pain efficacy, GS as a disease-modifying agent, and no side effects with sustained-release acetaminophen, the relative likelihood of continuing with sustained-release acetaminophen taken regularly is positive and approximately equivalent to GS. By contrast, the relative likelihood of continuing a selective or a non-selective NSAID, taken regularly, is negative. The cost sensitivity analysis for GS (Table 5) reveals that the predicted probability of continued use of GS when provided without charge is 91.6%, however when the cost rises to $50 per month, the predicted uptake drops to 75%.

**Table 5:** Cost sensitivity analyses for glucosamine sulfate

**Out of pocket monthly cost ($AUD)**	**Predicted probability of continued use (%)**
0	91.6
10	89.3
20	86.5
30	83.2
40	79.2
50	74.6

## Discussion

Using DCE, this study has assessed the factors that influence the decision to continue with a medication among a group of people with symptomatic OA. To the best of our knowledge, this is one of few DCEs assessing medication preferences nested within a clinical trial [46], and the first in an OA clinical-trial population. Such decisions underscore the concept of intentional medication non-adherence, which may influence the translation of clinical-trial results into practice.

This study has found that treatment factors, as opposed to respondent characteristics including self-reported pain levels and physical functioning, were driving adherence decisions. Preferences to continue with OA treatments were influenced by, in order of importance: the possibility of high blood pressure, heart/liver/kidney problems as side effects, out-of-pocket costs, the possibility of heartburn/reflux, or stomach ulcers as side effects, treatment schedule (i.e.: daily versus when required), mode of action (slowing OA versus symptomatic pain relief) and the possibility of drowsiness or constipation as a side effect.

Perhaps surprisingly, treatment efficacy did not significantly influence patient choices in this study. However in contrast to previous DCE studies conducted among OA patients in observational settings [23,24,28], this study included additional factors related to treatment schedule as well as cardiovascular, liver and renal side effects. When treatment schedule and cardiovascular, liver, and renal side effects were taken into account, as in the present study, their influence over patients’ treatment preferences then seem to dominate over considerations of treatment efficacy.

Assuming equal efficacy and GS as a disease-modifying agent, this study has found that the relative likelihood of continuing sustained-release acetaminophen and GS are positive and in contrast to NSAIDs. This disparity in predicted adherence was primarily driven by negative preferences expressed for cardiovascular, liver and renal side effects. This result may reflect an increased awareness of, or general aversion to, NSAID-related side effects through the recent and widely publicised removal of two NSAIDs, rofecoxib (2004) and lumaracoxib (2007), from the Australian market due to cardiovascular and hepatic toxicity, respectively [47,48].

However, the reduced likelihood of continuing NSAIDs compared to acetaminophen predicted from this study seems to be at odds with the high levels of self-reported use of NSAIDs in Australia by people with OA [3]. In light of the efficacy and improved safety of acetaminophen in OA compared to NSAIDs [3,5], our findings therefore reinforce the message that the uptake of guidelines-recommended acetaminophen in practice would benefit from ensuring that patient medication decision-making is supported through the provision of explicit risk/benefit information.

Medication adherence within a clinical trial is typically higher than in observational settings [49]. For the LEGS study, as the treatment was provided free of charge, the level of medication adherence observed in the trial was predicted to be higher than would exist outside of the trial setting. This was demonstrated in the findings of the cost-sensitivity analysis: when provided free of charge as per study protocol, the predicted continuation of GS is around 91%; however at the average current monthly price for GS ($20), this figure dropped to below 80%. Such rates of long-term adherence and their sensitivity to out-of-pocket costs will need to be factored into translation of the findings into policy and future economic evaluations.

The findings of this study must be viewed in light of its limitations. First, this study was conducted within a clinical-trial population, which may affect the generalisability of results. In particular, the self-reported adherence to medications and the proportion of participants using “when required” medications in this study was high. However, current medication use and self-reported adherence did not improve the model fit, suggesting that such preferences are formed independent of self-reported medication-taking behaviour.

Second, while discrete choice methods are widely used in health economics, an inherent limitation is that respondents are evaluating hypothetical medications; what respondents declare they will do may be quite different to what they would actually do if faced with the consequences of a choice. Forcing respondents to choose between medications may also be contrary to actual behaviour, particularly considering the over-riding influence of a prescriber’s recommendations upon patient preventive treatment decisions [22,50]. To minimise such potential differences, measures were taken to design the hypothetical tasks to be realistic, for instance by centring levels of cost on current treatment costs and describing pain on the same scale used throughout the clinical trial. Encouragingly, in this study, trust in prescribers and the actual self-reported adherence did not influence the model results. Nonetheless, to investigate the relationship between relative preferences captured in this study and one’s absolute preference to adhere to medications, future research incorporating the influence of the prescribers recommendation, for instance by allowing respondents to opt out of the non-adherent choice [31,50], is warranted.

Finally, as the constant term (α) was significant, the factors included in this study do not explain all of the behaviour modelled. Further work is therefore needed to clarify which other factors are being considered in adherence decisions.

## Conclusions

Osteoarthritis is a chronic condition incurring considerable costs to most health care systems. As with any chronic condition, non-adherence to the available pharmacological treatments is a problem that has the potential to impact on population health and expenditure. In the context of a clinical trial assessing therapy effectiveness, non-adherence has the potential to derail translation into clinical practice. By recognising that a component of this health behaviour is intentional and subject to rational choices, this study has characterised the complexity of medication-taking decisions for people with symptomatic OA that may lead to intentionally non-adherent behaviour, identifying the treatment factors driving such decisions. Such factors may be amenable to intervention such as strategic pricing. The salience of medication risks in such choices highlights the importance of providing appropriate risk/benefit information upon prescription. Cost was also a strong consideration in medication-taking decisions, a finding that ought to be acknowledged when interpreting clinical trial evidence for practice. Ultimately addressing these factors may be the way forward to realising the full potential health and economic benefits from the efficacious and safe use of osteoarthritis medications.

## Abbreviations

AIC: Akaike’s information criterion; DCE: Discrete choice experiment; GS: Glucosamine sulfate; LEGS: Long-term evaluation of glucosamine sulfate; LL: Log likelihood; MEDOS: Medication decisions in osteoarthritis study; MMNL: Mixed multinomial logit model; NSAID: Non-steroidal anti-inflammatory drug; OA: Osteoarthritis; OR: Odds ratio; PCAS: Primary care assessment survey; WOMAC: Western Ontario and McMasters universities arthritis index; WTA: Willingness to accept.

## Competing interests

The authors declare that they have no competing interests.

## Authors’ contributions

This work is a component of TL’s doctoral research supervised by SJ and JB. TL, SJ, MF and JB have all made substantial contributions to the conception, design, acquisition, analysis and interpretation of the data, as well to the critical revision of the manuscript. All authors have read and approved the final manuscript.

## Pre-publication history

The pre-publication history for this paper can be accessed here:

http://www.biomedcentral.com/1471-2474/14/160/prepub

## Supplementary Material

Additional file 1 LEGS participant eligibility criteria. Click here for file

Additional file 2 Discrete Choice Experiment Model Form and Analysis. Click here for file
