# Supplementary material for: Patient preferences for adherence to treatment for osteoarthritis: the MEdication Decisions in Osteoarthritis Study (MEDOS)
Source: BMC Musculoskelet Disord. 2013 May 6;14:160. doi: 10.1186/1471-2474-14-160 (PMC3660275; doi:10.1186/1471-2474-14-160)
Supplement: Additional file 1 — LEGS participant eligibility criteria. [file 1471-2474-14-160-S1.docx]

**Additional file 1**

LEGS participant eligibility criteria

*(see:* [*http://clinicaltrials.gov/ct2/show/NCT00513422?term=legs&rank=2*](http://clinicaltrials.gov/ct2/show/NCT00513422?term=legs&rank=2)*)*

| **Inclusion** |  |
| --- | --- |
| Age | 45-75 years |
| Symptomatic knee pain | Knee pain or taking NSAIDs/analgesia for pain on most days in the past month, |
|  | Knee pain 4-10 on a 0-10 numeric scale |
| Medial tibio-femoral compartment joint space ^1^ | Narrowing in symptomatic knee |
| **Exclusion** |  |
| Unstable diabetes |  |
| Medial tibio-femoral compartment joint space^1^ | Width <2mm |

1 Weight-bearing magnification-controlled semi-flexed radiographs were taken of both knees using the strictly standardised metatarsophalangeal protocol and foot maps [42]. To be eligible, a sufficiently symptomatic knee needed to demonstrate reduced medial tibio-femoral compartment joint space width, compared with the lateral compartment, but retain at least 2.0mm minimum medial joint space width.
