# Supplementary material for: Patient preferences for adherence to treatment for osteoarthritis: the MEdication Decisions in Osteoarthritis Study (MEDOS)
Source: BMC Musculoskelet Disord. 2013 May 6;14:160. doi: 10.1186/1471-2474-14-160 (PMC3660275; doi:10.1186/1471-2474-14-160)
Supplement: Additional file 2 — Discrete Choice Experiment Model Form and Analysis. [file 1471-2474-14-160-S2.docx]

**Additional File 2:**

**Discrete Choice Experiment Model Form and Analysis**

The model assumed that the relationship between the levels of observed out-of-pocket cost and pain efficacy of each alternative and the corresponding weights were linear and corresponded to the following form:

*U_isj_ = α_is_+β*_1_**(pain efficacy) +β*_2_**(mode of action) + β*_3_**(dose frequency) + β*_4_**(treatment schedule) + β*_5_**(out of pocket monthly cost) + β*_6_**(prescription) + β*_7_**(side effect-drowsy) + β*_8_**(side effect-gastrointestinal) + β*_9_**(side effect-heart/liver/renal) + ε_isj_*

where *β_(1-9)_* are the associated parameter estimates or relative weights for each factor. An alternative specific constant (α) was specified to represent the mean of the distribution of the unexplained effects. *β_(2,3,4, 6,7-9)_* were effects coded and relative to the base level of each corresponding factor indicated in Table 1. The parameter estimates describe the magnitude of utility change and indicate the relative impact of a unit change of a factor to the decision. *ε_isj_* is the random or non-explainable error term associated with each choice set and accounts for unobserved preference variation, specification error, or measurement error [32, 38].

All β parameters were initially treated as random with normal distribution except cost, which was initially treated as random with a constrained triangular distribution. Models were evaluated for goodness of fit using McFadden’s pseudo R^2^ and Akaike’s information criterion (AIC). The log-likelihood (LL) ratio test was used to determine model significance against each other and for overall model significance. Final analyses were conducted using 1000 Halton draws from the random parameters distribution, which accelerates the estimation by 5-10 compared to simple pseudo-random draws [38].
